# Differences in visio-spatial intelligence between non-athletes and netball players

**DOI:** 10.3389/fspor.2023.1109967

**Published:** 2023-02-20

**Authors:** Nonkululeko Mathe, Lourens Millard, Gerrit Jan Breukelman, Musa Mathunjwa

**Affiliations:** Department of Human Movement Science, University of Zululand, Kwadlangezwa, South Africa

**Keywords:** vision in sport, visio-spatial intelligence (VSI), visual skills, netball vision, sport vision

## Abstract

There is conflicting evidence regarding whether athletes have better visio-spatial skills than non-athletes. This gap may result from athletes' superiority in only some visio-spatial abilities (VSS), rather than all areas of vision. The aim of this study was to determine whether there is a significant difference in the visio-spatial intelligence between female netball players (*n* = 40) and non-athletes (*n* = 40) when comparing six visual skills (accommodation facility, saccadic eye movement, speed of recognition, peripheral awareness, hand-eye coordination, and visual memory). Following an optometric evaluation, the participants were assessed in six distinct established tests, including the hart near far rock, saccadic eye movement, evasion, accumulator, ball wall toss tests, and flash memory, to evaluate the VSS components of non-athletes and premier league netball players. For five of the six tests, there was a statistically significant (*p* ≤ 0.05) difference between netball players and non-athletes. Conversely, there is no concrete evidence that netball players have better visual memory than non-athletes (*p* = 0.277). When compared to non-athletes, netball players have significantly improved accommodation facility (*p* < .001), saccadic eye movements (*p* < .001), speed of recognition (*p* < .001), peripheral awareness (*p* < .001), and hand-eye coordination (*p* < .001), but not visual memory (*p* = 0.277). The findings that netball players perform better on a certain VSS have broad ramifications for theories of sport vision, the optimal way to choose tests, and the creation of VSS testing batteries for specific sports.

## 1. Introduction

Top-level sports performance in stressful and challenging situations necessitates the optimal functioning of a variety of cognitive skills such as decision making, attention and working memory (1). According to Vestberg et al. (2) improved brain functioning in male and female athletes may improve performance and predict competitive success. This cognitive functioning is thought to be especially important in open sports that require constant attention, management of multiple factors, or adaptation to changing settings (3). The visual motor system makes use of three primary ocular motor skills: vergence which is the coordinated movement of the eyes' pupils during focusing, either toward or away from one another; focusing (to eliminate irrelevant background information and concentrate on crucial visual information) and tracking (a visual processing ability that takes place when the eyes fixate on a moving object in the field of vision). Wilson and Falkel (1) state that the eyes must be able to converge (or cross) as the ball comes toward the player and uncross (or diverge) when the ball moves away from the player.

In the past, classification, and comparison of athletes to non-athletes have depended on physical condition assessment and training methods (4). However, there is now growing interest in how testing and training for vision might influence sports performance and help differentiate between athletes and non-athletes (5, 6). According to Gao et al. (7), athletes may have better vision than the general population. However, because different components of vision are used in different sports and because there are various visual skills, athletes can develop their visual abilities through practice and assessment (8, 9). Visio-spatial skills (VSS) are a broad category of specialized abilities that range from recognizing light and dark, recognizing intricate intersecting angles and curves, to recognizing faces from the features of their hair, noses, lips, and eyes (10, 11). and is an essential element of coordination in everyday living and across all sporting codes.

Netball can be categorized as non-static sport due to the visual information changing and needing to be processed continuously for longer than an hour, which requires visual attention, and dynamism for the player to keep processing game-based information (12). According to Abernethy (13), most of the visual testing and training programs tend to concentrate primarily on the fundamental visual functions, failing to recognize the necessity for sport-specific testing and training to be effective and increase on-field performance.

In the past, studies on netball players have mostly concentrated on physiology, performance enhancement, injuries, and masculinity (14–17). Limited research exists on netball players' visio-spatial intelligence, especially with relation to a sport specific test battery, with the majority focusing on multiple team sports and recreation (18–20). However, it has been proved that VSS can improve brain function when stimulated appropriately (21), and non-athletes use their VSS daily to carry out activities, yet they are frequently inactive, which might cause a decline in several VSS (22). In this vein, it is critical to identify which VSS are amplified in netball players, if any, because there is conflicting information on VSS in athletes vs. non-athletes.

## 2. Aims and hypothesis

The aim of this study was to discern whether there is a significant difference in the visio-spatial intelligence between netball players and non-athletes. In this study, six VSS components including accommodation facility, saccadic eye movement, speed of recognition, peripheral awareness, hand-eye coordination, and visual memory were examined between netball players and non-athletes. The hypothesis is as follows: The visio-spatial intelligence will be significantly higher in netball players when compared to non-athletes. There is a definite and clear lack of research with regards to using sport specific test batteries when testing athletes (18–20). For the purposes of this study a proven visio-spatial intelligence test battery was employed and a study using such a test battery on netball players, and then comparing the results to non-athletes seems crucial (18).

## 3. Methods

### 3.1. Participants

The study sampled (*N* = 80) 18- to 27-year-old females. The participants were divided into non-athletes (*n* = 40) who have not participated in any form of sport, and female premier league netball players (*n* = 40) (Table 1). One requires a lot of participants to get the richness of data or the significant difference necessary for qualitative analysis (at least 30 to 60) (23). It was decided to test 40 participants since that's the number of netball players that were available. Since research indicates that there is a VSS difference between males and females, it was decided to only compare females to one another so that sex differences could not influence the results (24). After institutional ethics approval and informed consent. Non-athletes were recruited from the Zululand region of South Africa's KwaZulu-Natal province and were not required to participate in any structured sporting/physical activity program (25). A non-probability sampling strategy was used to choose netball players from all premier league netball teams in the same geographic area as the non-athletes. Players in the Premier League are required to have participated in at least one competitive match per week for the previous three months and have completed 30 h of netball training. Age matching was done among participants in each group to maintain balance. Institutional ethics approval date and number (UZREC 171110-030 PGM 2022/55).

**Table 1:** Comparing descriptive information of netball players and non-athletes.

	Non-athletes (*n *= 40)	Netball players (*n *= 40)
Age (Yr)	22.13 ± 2.37	20.58 ± 2.21
Height (cm)	169 ± 0.09	179 ± 0.07
Weight (kg)	64.51 ± 7.39	70.88 ± 8.43
Years of experience (Yr)		6.98 ± 3.01

Mean ± SD; Years (Yr); Centimeters (cm); Kilogram (kg).

### 3.2. Optometric assessment

The Spectrum Eyecare program (Version 6.0.0, Digital Optometry, Republic of South Africa) was used to conduct a general optometric screening on each participant to ascertain whether any visual impairments prevented them from participating in the study. All participants claimed to have normal or corrected-to-normal 20/20 vision, only 2 netball players wore glasses normally for corrective vision, but used contact lenses during the testing to ensure playing conditions (as they use contact lenses in matches and practice sessions) were simulated. None of the participants had ever taken a VSS test before.

### 3.3. Visio-spatial skill test battery

To reduce the impact of any food or supplement influences, testing was performed in the post-absorptive state after a 9 to 12-hour fast on weekday mornings between 07:00 and 12:00. Participants were only evaluated 48 h after participating in any physical activity to minimize any physical and psychological consequences. All participants were verbally questioned to ensure they had followed the dietary requirements. The same VSS test battery was administered to both groups, and tests were run in the order listed below to prevent the influence of earlier tests on subsequent test: Accommodation facility, saccadic eye movements, speed of recognition, hand-eye coordination, peripheral awareness, and visual memory. To ensure appropriate recovery, a five-minute rest interval was observed in between each session.

### 3.4. Strengths and limitations

The study had several strengths. It gave an in-depth look at the visual abilities needed for netball and the visual differences between athletes and non-athletes, the study described in detail the design of the testing protocol and how it was performed, for replication purposes. For the purposes of the study, participants were picked in accordance with the research design. The study appropriately conveys the data's statistical significance that was found between the two groups, which is novel in nature specifically with relation to netball players. Sample size is a possible limitation, as there was only a limited amount of netball players in the region which was available and willing to be part of the study. There are multiple factors affecting vision, and even though this was taken into account when testing and creating the protocol, there is always the possibility of factors outside the parameters of control having an influence on the tests.

#### 3.4.1. Accommodation facility

A Hart chart was used to assess an eye's capacity to locate and identify an item at a certain distance, as well as its position, orientation, and refractive errors (26, 27). The test has a reliability of 0.7, making it a legitimate and reliable test to use (18, 28). On a wall three meters away, the Hart Chart was positioned at each participant's head height. A second chart had to be held by the participants at arm's length using their dominant hand, the paper was held perpendicular to the body at shoulder height in the frontal plane and their hands had to be stationary. The participants were then requested to read aloud the first letter of the first line of the chart from a three meters distance, and they were then told to read aloud the first letter of the chart from an arm's length away (28, 29). After 30 s, the participants were instructed to continue in this manner, at which point their errors and final score were recorded (i.e., how many letters were read correctly). The participant's number of errors was deducted from the final score to arrive at the final score. To verify that letters cannot be memorized, various charts were utilized (26, 28). A five-minute break was taken between each session, and the highest score from the two trials was recorded and applied in the analysis. This test was time based the participants had to score as high as possible in the allocated time.

#### 3.4.2. Saccadic eye movements

A saccadic eye movement chart was used to measure how quickly the participant's eyes travel when moving the fovea to a new spot in the visual surroundings (1, 30, 31). This test has a reliability of 0.703 (28). On both sides of the page, this chart included movable letters that ran vertically downward. To prevent letters from being memorized, various charts were utilized (28). The subject stood 3 m away from two saccadic eye movement charts that were mounted on a wall and spaced apart by 1 m. The subject was then required to quickly turn to the right chart and read aloud the first letter on the lateral side before doing the same on the left chart's lateral side. The participants were told to just move their eyes and to keep their heads steady. The duration of the test was 30 s, and both the final score and the number of faults were recorded (i.e., how many letters were read). By deducting the number of mistakes from the total number of letters read, the final score was determined. The highest score from the two trials—each with a five-minute rest period between them was recorded and used in the analysis (28). This test was time based the participants had to score as high as possible in the allocated time.

#### 3.4.3. Speed of recognition

A Batak Pro (Quotronics, Surrey, United Kingdom) was used to measure the participant's speed of recognition (32), which has a reliability of 0.946 and requires the subject to react to a mobile stimulus (28). The Batak pro equipment is distinguished by its ability to emit light with a lifespan of one second, which is utilized to trigger a neurological speed of response in test subjects in sports such as tennis, basketball, rugby, and netball as well as non-athletes (18). In addition to the “scoring” LED screen displaying each successful strike, the Batak's “time” LED panel counts down the targets from 100 to zero, this software allowed 100 timed targets to glow at random. The targets were kept lit for a possible strike window of one second. The entire procedure accelerated if the incorrect target was hit, or if it was hit “out of time.” The subject was instructed to avoid hitting any flashing targets while performing this exercise. The athlete received a verbal “foul” from the apparatus and lost five points for hitting the target. The infrared beam was released, and the participant had to dodge it instantly to avoid losing five points if all the center targets lit up simultaneously. The device kept track of the mistakes made, and after automatically subtracting them from the final score, it calculated the score (32). The highest score from the two trials was recorded and used in the analysis after a five-minute break between each session. The maximum score for this test was 100 points.

#### 3.4.4. Peripheral awareness

The Accumulator Program of the Batak Pro (32) was used to measure participants' capacity to see things and motion that are not directly in front of them. This test has a reliability of 0.885 (18). As part of the Accumulator Program, random targets on the Batak Pro remained lighted for 60 s before the participant touched them with their hands (32). Two trials were conducted with a five-minute break in between, and the system recorded the targets that were successfully touched in 60 s. The top score was then noted and used to the analysis's final stage. This test was time based the participants had to score as high as possible in the allocated time.

#### 3.4.5. Hand-eye coordination

The current study used the tennis ball wall throw to evaluate hand-eye coordination (33). This test has a reliability of 0.708 (28). Each participant had to toss the ball at the wall from a point that was indicated 2 meters away and catch it with their alternate hands, they started tossing the ball using their non-dominant hand first. This was done by participants for 30 s (28). Scores were tallied for each ball that the participant successfully caught. Within the allotted 30 s, the participants had to complete as many catches as they could. The highest score from the two trials were recorded and used in the analysis after a five-minute break between each session. This test was time based the participants had to score as high as possible in the allocated time.

#### 3.4.6. Visual memory

To assess the memory from which the visual system received the stored information, six randomly chosen targets were flashed for 0.5 s using the Batak Pro Flash Program (28). The reliability of this test is 0.735 (28). Participants had to correctly recall the individual targets and the order in which they were triggered after hearing the “double beep” instruction to strike the lighted targets (which lit up in a specific order). There was a maximum number of fifty-four targets the subject could strike, and with every incorrect target struck, this score would decrease. The “score b” LED panel showed the maximum score, and the “score a” LED screen indicated the points scored for each successful strike (32). The number of successful strikes was then recorded. In the final data analysis, the highest score from the two trials was recorded and used. Between trials, there was a five-minute rest period. A possible highest score for this test is 54 points.

### 3.5. Data analysis

The study made use of pre-existing VSS evaluations and quantitative research methods. Using the Statistical Package for Social Sciences (SPSS) version 22 for Windows (IBM Corporation, Armonk, NY, United States) the descriptive statistics for the data collected, including standard deviations, means, percentage difference and ranges, were assessed for this study. The Mann-Whitney U test was used to assess differences between the two independent groups because the dependent variable was continuous and not regularly distributed. To confirm the Mann–Whitney *U* test findings and determine which netball players had the more superior visio-spatial skills, a rank-ordered analysis was conducted. This was done in order to establish the significance of differences that exist between the results of the specific tests, to determine which visual skill and test showed the highest significant difference. Statistical significance was set at *p* ≤ 0.05.

## 4. Results

The findings showed that there was a significant difference (*p* ≤ .05) between netball players and non-athletes in five of the six tests (Table 2). In terms of accommodation facility (*p* < .001), saccadic eye movement (*p* < .001), speed of recognition (*p* < .001), peripheral awareness (*p* < .001) and hand-eye coordination (*p* < .001) netball players significantly outperformed non-players, but not in terms of visual memory (*p* = .277).

**Table 2:** Visio-motor expertise comparison in netball players and non-athletes.

Visio-spatial skill	Non-athletes (*n *= 40)	Netball players (*n *= 40)	Difference (%)	Significance (*p*-value)
Accommodation Facility	28.78 ± 2.9	35.35 ± 6.5	19	<.001
Saccadic Eye Movement	32.98 ± 4.7	44.70 ± 9.8	26	<.001
Speed of Recognition	11.23 ± 6.1	24.70 ± 16.1	55	<.001
Peripheral Awareness	56.20 ± 3.4	63.35 ± 6.4	11	<.001
Hand-Eye Coordination	19.55 ± 4.5	23.40 ± 4.8	16	<.001
Visual Memory	45.10 ± 5.5	43.83 ± 4.9	3	.277

Mean ± SD; Statistically significant (*p* ≤ .05).

Netball players performed 55% faster at speed of recognition than non-athletes, according to a rank-ordered analysis, followed by 26% for saccadic eye movement, 19% for accommodation facility, 16% for hand-eye coordination, and 11% for peripheral awareness (Table 1).

## 5. Discussion

The aim of this study was to discern whether the visio-spatial intelligence is higher in netball players in comparison to non-athletes. In this study, six VSS components including accommodation facility, saccadic eye movement, speed of recognition, peripheral awareness, hand-eye coordination, and visual memory were particularly examined between netball players and non-athletes.

The current study discovered that the accommodation facility is higher in netball players when compared to non-athletes. This is comparable to studies by Jafarzadehpur et al. (34) and Gao et al. (7) where the female volleyball players showed better facility of accommodation than non-athletes. However, Ripoll & Latiri (35) found that accommodative facility did not differ significantly in this regard. For ball interceptions, control, passing, and throwing as well as analyzing the positions of teammates and opponents, among other things, near/far visual alterations are constantly occurring (36). These actions encourage the vergence/accommodative system's continual involvement, which may have effects like those of visual therapy exercises (37). Some athletes were concluded to have a lower amplitude of accommodation than non-athletes, suggesting that they are only superior to non-athletes when they focus on multiple targets rather than single targets which may explain why no increase in accommodation facility was found in the studies of Zwierko et al. (38). Accommodation facility allows athletes to quickly shift their attention to follow the ball while sustaining visual clarity.

This study agrees with the study by Zwierko et al. (39) where soccer players showed better saccadic eye movements than non-athletes. The study by Babu, Mellers and Irving (40) showed that the athletes did not respond better in terms of accuracy of saccades compared to non-athletes. Athletes need to suppress prosaccades strongly and prepare for voluntary events more quickly to do this (41). Training shortens the antisaccade production phase, according to Vera et al. (42). Abernethy (13), on the other hand asserts that visual training is unable to enhance either motor or fundamental visual function. However, Maman, Gaurang and Shadu (43) revealed that visual training improves visual functions, including saccadic eye movements. Yilmaz and Polat (44), hypothesized that training and having a greater visual attention level may be crucial factors in an athlete's ability to suppress reflexive prosaccades. For this reason, experienced athletes show more effective gaze strategies than novice athletes (45). The study by Babu, Mellers and Irving (40) showed that the athletes did not respond better in terms of accuracy of saccades compared to non-athletes which was possibly due to poor spatial organization skills caused by a lack of controllability in monitoring factors that effects visual skills like caffeine intake. A smooth tracking movement or a quick hop may be required when the athlete quickly scans from one sight to the next.

The finding of speed of recognition in netball players and non-athletes in this study is consistent with that of Millard et al. (18), where they demonstrated that rugby players were superior to non-athletes, and Zwierko (46) who found better speed of recognition skills when comparing handball players to non-athletes. The current study discovered that the largest difference between netball players and non-athletes (in that netball was visio-spatial scores were higher for netball players when compared to non-athletes) was found in speed of recognition, with netball players scoring 55% higher than non-athletes. According to Kaya (47), decision-making in the world of sport exhibits three features. First off, since decision-making is naturalistic, athletes naturally confront it in a sport context with some level of task familiarity. Additionally, if the test battery is sport-specific, athletes will do better than non-athletes because they have improved decision-making abilities when presented with a visio-spatial decision that frequently arises in their sport (47). Lastly, most sports decisions are dynamic, thus they usually become clearer with time. The dynamic feature of decision-making has a double impact, which means that information is not instantaneously gathered and processed, but rather a decision-maker must accumulate knowledge over time, and subsequent processing of this information takes additional time. Sports-related decisions, however, include external dynamics, which means that the circumstance itself can change over time (47). In addition, most decisions made by athletes are made while they are moving, which makes them more difficult to make. Speed of recognition may be the most important VSS associated with netball success since decision making in sport is realistic, dynamic, and executed in motion (which raises the difficulty level). To make play-related choices like whether to pass the ball, where to position oneself next, and other things, players need to be able to process a lot of information with just a fast sweep of the court.

Several studies have shown that athletes have better peripheral awareness and the ability to track a moving target, as well as a different strategy for processing visual information than non-athletes (48–50). Ciućmański and Wątroba (51) found that 12-year-old football players performed better than their non-athletic classmates in tests of depth perception, peripheral vision, and the capacity to visually track a moving object. It was found that visual perception training raised the levels of these skills and, as a result, improved the effectiveness of an athlete's perception. However, football players aged 9 to 17 with varying degrees of experience and non-athletes had their peripheral vision tested by Ward and Williams (52). Although they noticed an increase in visual function with aging, expert soccer players did not perform noticeably better than players with less athletic background. Furthermore, none of the groups under comparison demonstrated visual abilities that were above average compared to the overall population. In situations like those that demand watching a ball and numerous players in order to process essential information, peripheral vision will likely be used extensively, and attention will be distributed proportionally to various locations.

The findings of this study can be compared with those of Venter and Ferreira (53), who evaluated rugby players of various ages on their visual skills and had better hand-eye coordination compared to non-athletes. The authors anticipated that age and associated motor development could have an impact on visual perception abilities. Moreso, Kioumourtzoglou et al. (54) found a comparable result in members of a Greek national team, arguing different perceptual strategies from experts to novices in relevant and irrelevant cues. The fact that people with inherent neurological advantages like eye-hand reaction time and visuospatial intelligence can easily participate in sports and the fact that exercise is good for eye-hand reaction time and visio-spatial intelligence are two explanations for the athlete's advantages in both areas (21). To sprint, jump, catch, change directions, push, shoot, and block, players must master high-level of eye body coordination.

The only VSS out of the six assessed that was shown to be comparable between netball players and non-athletes was visual memory. A study by Tomczyk et al. (55) and this study's results are comparable. Another study compared the visual memory capacity of non-athlete college students to that of team sport athletes and found no significant differences (56) but researchers also examined exercise's potential to enhance visual memory, the capacity to actively monitor, alter, and interpret fresh information. These studies have validated the idea that more engagement in sports is linked to improved visual memory abilities, much like exercise (57, 58). Our contradictory result may have several explanations, but one of them is that while individual differences in naturalistic exercise habits do not correspond to noticeable between group differences in visual memory, improvements in visual memory have been shown in previous studies to occur relative to each participant's baseline skills in response to the experiment's increased levels of physical activity (56). Players will be able to make rapid and accurate decisions about what to do next in a game by employing visual memory with the aid of the visuals they record.

The findings of this research demonstrated that netball players surpassed non-athletes in VSS performance. There is evidence that during visio-spatial demands, athletes develop unusual mechanisms of occipital brain synchronization, demonstrating superior visio-motor performance to non-athletes (59). It is crucial to recognize the significance of a fortuitous genetic tendency towards an increased VSS in sportsmen. Components of athletic performance like power, endurance, flexibility strength, neuromuscular coordination, psychological qualities, and other phenotypes are greatly influenced by genetic variables. Athlete status is therefore a heritable trait, with additive genetic variables accounting for an average of 66% (depending on the athletic discipline) of the variance in athlete status. Non-shared environmental factors are responsible for the remaining variation (60). Sports vision training methods and consistent practice help to enhance specific visual skills.

The current study's hypothesized that netball players would have better VSS than non-athletes, based on previous research. This was supported by the fact that this study found that netball players had 55% higher speed of recognition skills than non-athletes, followed by 26% for saccadic eye movement, 19% for accommodation facility, 16% for hand-eye coordination, and 11% for peripheral awareness. These results imply that these VSS may be required in varying degrees of importance for netball performance. To ascertain whether the level of play or some other variable may be associated to VSS in athletes and/or for a certain sport, it is also important to record and correlate the level of expertise of the netball players (i.e., the number of years of practice).

It is crucial to comprehend how the visual system works during sport performance in order to decide whether, when, and how sportsmen and sportswomen might be supported to improve their skill performance through vision-based intervention programs (61). Analog “eye fitness” activities were once employed as the first methods of sport visual training (62). These challenging drills forced students to rapidly switch from convergence, accommodation, saccadic, and/or pursuit eye movements to visual targets, placing great demands on the oculomotor system. Using the same set of analog vision training activities over a five-week period, it was shown that individuals who got vision training exhibited faster and more accurate netball passing compared to the control group (62).

## 6. Conclusion and practical implications

The finding that only a few VSS are superior to others has far-reaching implications for vision theories such as theories of visual perception (direct, indirect, and computational), the best way to select tests, and the development of sport specific VSS testing batteries. While this study supports the idea that athletes, particularly netball players, have better VSS than non-athletes, this study also found that this is not the case with all VSS, as with visual memory in this study. Similarly, to how anthropometric and physiological tests are used in athlete selection and recruitment, research should be conducted to determine which VSS tests are more capable of distinguishing athletes from non-athletes. Beginners and other netball players who could require greater visual performance may be considered to need visual training. Future research should examine how much visual perceptual learning and physical fitness influence the findings now seen in elite athletes.

## Data Availability

The raw data supporting the conclusions of this article will be made available by the authors, without undue reservation.
